# Supplementary material for: CRISPR/Cas14a combined with RPA for visual detection of Marek’s disease virus
Source: Microbiol Spectr. 2026 Feb 6;14(3):e02625-25. doi: 10.1128/spectrum.02625-25 (PMC12955468; doi:10.1128/spectrum.02625-25)
Supplement: Supplemental figures — Figures S1 to S4. [file spectrum.02625-25-s0001.pdf]

Supplementary Figure

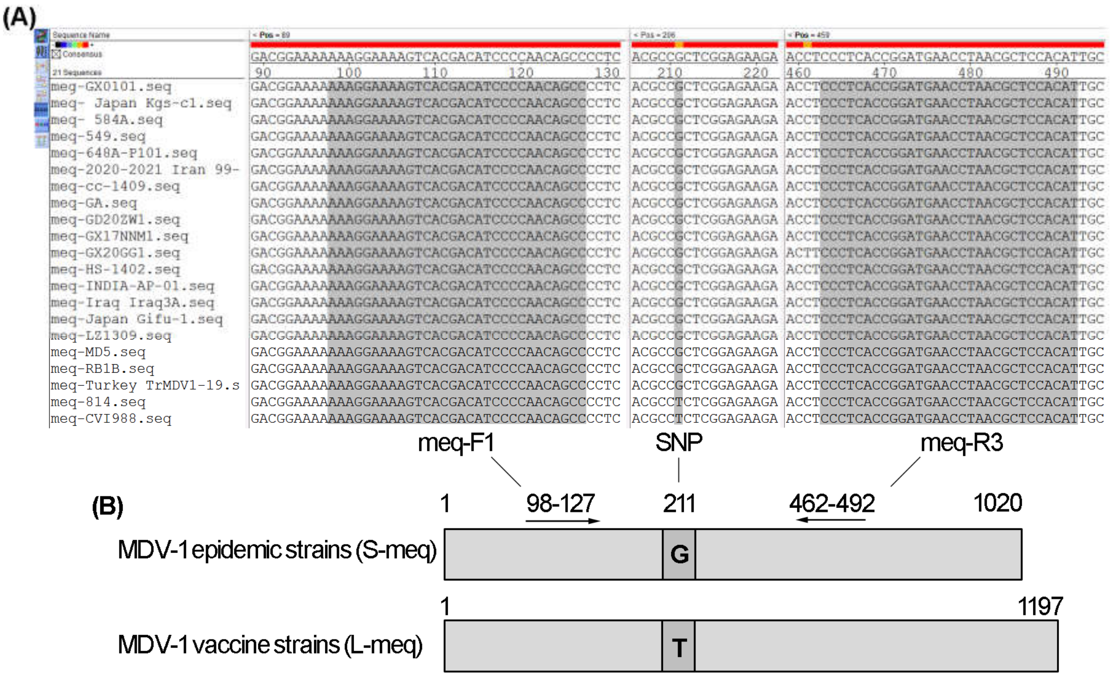

Fig. S1: Schematic diagram illustrating the conservation analysis of the meq gene sequence among MDV-1 reference strains and the design of RPA primer pairs. A, the upstream and downstream primers, meq-F1 and meq-R3, target specific conserved regions of the meq gene across different pathogenic MDV-1 reference strains; SNP, denotes a characteristic single nucleotide polymorphism within the amplified fragment, enabling specific differentiation between MDV-1 field and vaccine strains. B, shows the relative binding positions of the upstream and downstream primers on the meq gene; "→" and "←" represent the upstream and downstream primers, respectively; numbers indicate relative nucleotide positions; S-meq refers to the relatively shorter meq gene variant found in MDV-1 field strains; L-meq refers to the longer meq gene variant present in MDV-1 vaccine strains.

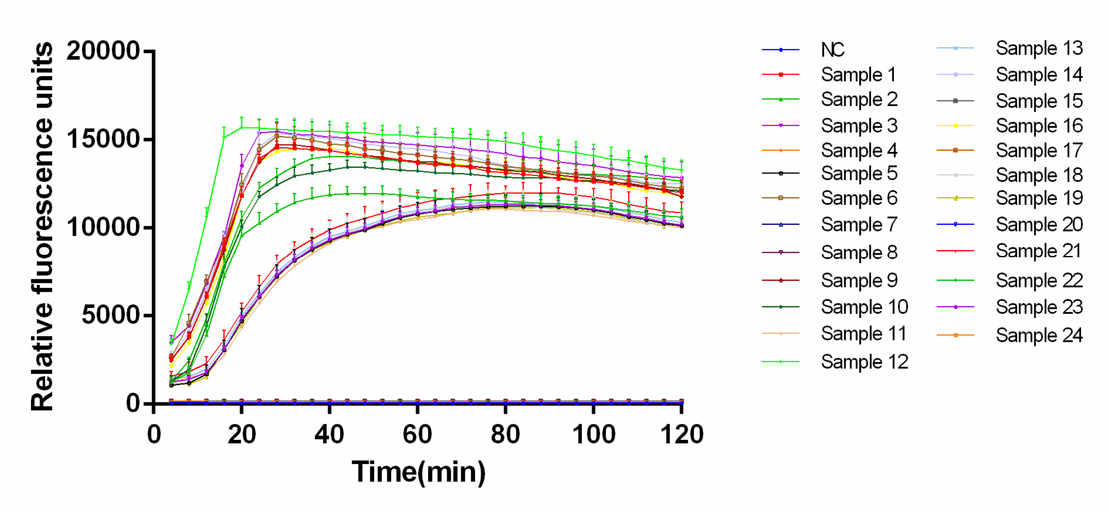

Fig. S2: Detection of virus in clinical samples using an RPA-CRISPR/Cas14a-based fluorescence assay.

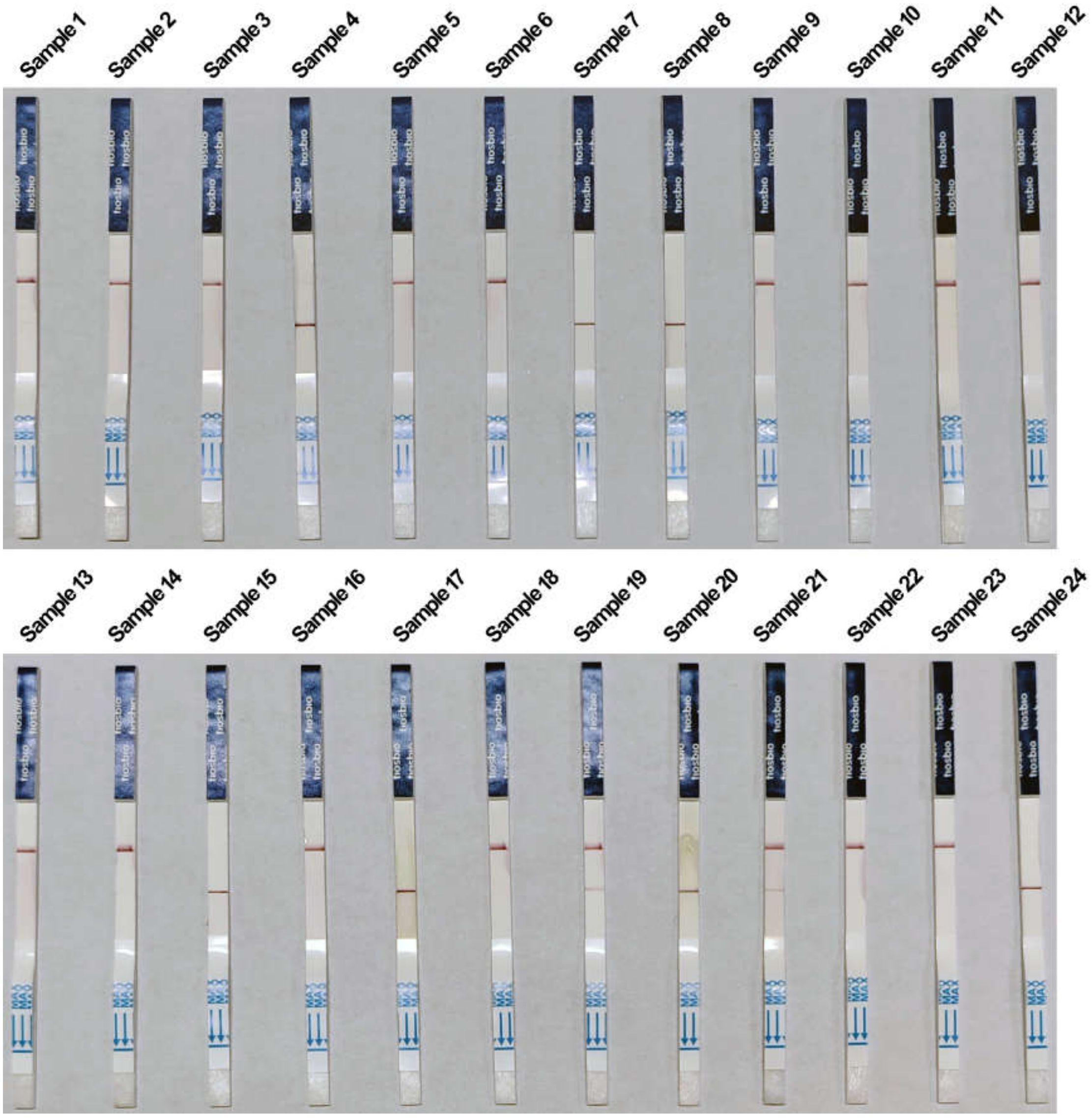

Fig. S3: Detection of virus in clinical samples using an RPA-CRISPR/Cas14a-based lateral flow

assay.

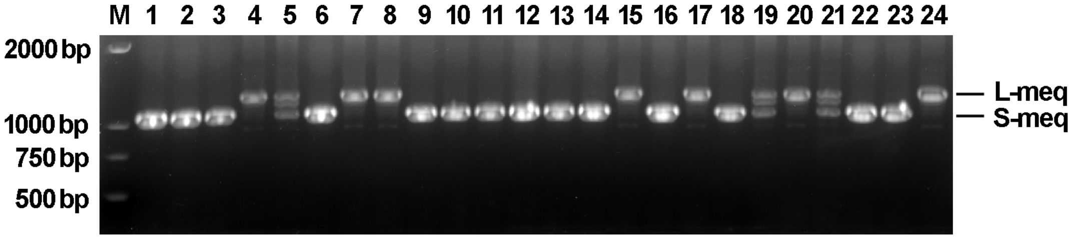

Fig. S4: Detection of virus in clinical samples by conventional PCR. Agarose gel electrophoresis image shows the PCR amplification products from clinical samples. M: marker; 1: sample 1; 2: sample 2; 3: sample 3; 4: sample 4; 5: sample 5; 6: sample 6; 7: sample 7; 8: sample 8; 9: sample 9; 10: sample 10; 11: sample 11; 12: sample 12; 13: sample 13; 14: sample 14; 15: sample 15; 16: sample 16; 17: sample 17; 18: sample 18; 19: sample 19; 20: sample 20; 21: sample 21; 22: sample 22; 23: sample 23; 24: sample 24.
