# Supplementary material for: CRISPR/Cas14a combined with RPA for visual detection of Marek’s disease virus
Source: Microbiol Spectr. 2026 Feb 6;14(3):e02625-25. doi: 10.1128/spectrum.02625-25 (PMC12955468; doi:10.1128/spectrum.02625-25)
Supplement: Table S1 — Viral loads in clinical samples as determined by qPCR targeting the pp38 gene. [file spectrum.02625-25-s0002.docx]

TABLE S1 Viral loads in clinical samples as determined by qPCR targeting the *pp38* gene

| **Sample** | **Ct** | **Copies*** | **Cas14a** **detection** |
| --- | --- | --- | --- |
| NO.1 | 24.36 | 1.17×10^5^ | Positive |
| NO.2 | 25.64 | 6.15×10^4^ | Positive |
| NO.3 | 27.85 | 1.5×10^4^ | Positive |
| NO.4 | / | / | Negative |
| NO.5 | 28.87 | 5.2×10^3^ | Positive |
| NO.6 | 23.92 | 1.55×10^5^ | Positive |
| NO.7 | / | / | Negative |
| NO.8 | / | / | Negative |
| NO.9 | 24.54 | 1.25×10^5^ | Positive |
| NO.10 | 25.78 | 7.13×10^4^ | Positive |
| NO.11 | 28.36 | 1.14×10^4^ | Positive |
| NO.12 | 21.28 | 1.3×10^6^ | Positive |
| NO.13 | 26.54 | 1.99×10^4^ | Positive |
| NO.14 | 24.21 | 1.63×10^5^ | Positive |
| NO.15 | / | / | Negative |
| NO.16 | 24.33 | 1.21×10^5^ | Positive |
| NO.17 | / | / | Negative |
| NO.18 | 27.44 | 1.06×10^4^ | Positive |
| NO.19 | 29.42 | 5.36×10^3^ | Positive |
| NO.20 | / | / | Negative |
| NO.21 | 27.63 | 8.71×10^3^ | Positive |
| NO.22 | 25.08 | 7.82×10^4^ | Positive |
| NO.23 | 23.93 | 1.96×10^5^ | Positive |
| NO.24 | / | / | Negative |

Note: “*” Number of copies of virus per million cells
